# Spatial and neighborhood data in the collaborative cohort of cohorts for COVID-19 Research (C4R)

**DOI:** 10.1371/journal.pone.0352170

**Published:** 2026-07-22

**Authors:** Jana A. Hirsch, Lilah M. Besser, Marcia Pescador Jimenez, Stephen T. Dickinson, Talea Cornelius, Stephen T. Francisco, Katherine Lawin, Hoda S. Abdel Magid, Sandra S. Albrecht, Norrina Bai Allen, Pallavi Balte, Sharrelle Barber, Lori A. Bateman, Lauren B. Beach, Jason P. Block, Scott C. Brown, Russell G. Buhr, Earle C. Chambers, James L. Crooks, Ana V. Diez Roux, Mitchell S. V. Elkind, Linda C. Gallo, Penny Gordon-Larsen, Jose Gutierrez, Carmen R. Isasi, Peter James, Suzanne E. Judd, Alka M. Kanaya, Namratha R. Kandula, Joel D. Kaufman, Kiarri N. Kershaw, Anna M. Kucharska-Newton, Erin R. Kulick, Joyce S. Lee, Gina S. Lovasi, Dave Mauger, Leslie A. McClure, Steven Melly, Sharon Stein Merkin, Yvonne L. Michael, Gabriela R. Oates, Brenda R. Phillips, Jessica A. Reese, Elizabeth A. Regan, Cameron J. Reimer, Daniel A. Rodriguez, Tatjana Rundek, Michael Schembri, Mario Sims, Nicole L. Spartano, Carla Wilson, Yiyi Zhang, Elizabeth C. Oelsner

**Affiliations:** 1 Urban Health Collaborative and Department of Epidemiology and Biostatistics, Dornsife School of Public Health, Drexel University, Philadelphia, Pennsylvania, United States of America; 2 Department of Neurology, Miller School of Medicine, University of Miami, Miami, Florida, United States of America; 3 Department of Epidemiology, Boston University, Boston, Massachusetts, United States of America; 4 Urban Health Collaborative, Dornsife School of Public Health, Drexel University, Philadelphia, Pennsylvania, United States of America; 5 Center for Behavioral Cardiovascular Health, Columbia University Irving Medical Center, New York, New York, United States of America; 6 Department of Medicine, Columbia University Irving Medical Center, New York, New York, United States of America; 7 Department of Population and Public Health Sciences and Spatial Sciences, University of Southern California, Los Angeles, California, United States of America; 8 Department of Epidemiology, Mailman School of Public Health, Columbia University, New York, New York, United States of America; 9 Institute for Public Health and Medicine, Feinberg School of Medicine, Northwestern University, Chicago, Illinois, United States of America; 10 The Ubuntu Center on Racism, Global Movements & Population Health Equity and Department of Epidemiology and Biostatistics, Dornsife School of Public Health, Drexel University, Philadelphia, Pennsylvania, United States of America; 11 Department of Biostatistics, Gillings School of Global Public Health, The University of North Carolina, Chapel Hill, North Carolina, United States of America; 12 Medical Social Sciences, Northwestern Feinberg School of Medicine, Northwestern University, Chicago, Illinois, United States of America; 13 Department of Population Medicine, Harvard Pilgrim Health Care Institute, Harvard Medical School, Boston, Massachusetts, United States of America; 14 Department of Public Health Sciences, Miller School of Medicine, University of Miami, Miami, Florida, United States of America; 15 David Geffen School of Medicine & Fielding School of Public Health at UCLA, Los Angeles, California, United States of America; 16 Department of Family and Social Medicine and Department of Epidemiology & Population Health, Albert Einstein College of Medicine, New York, New York, United States of America; 17 Division of Biostatistics and Bioinformatics, National Jewish Health, Denver, Colorado, United States of America; 18 Department of Neurology, Vagelos College of Physicians and Surgeons, and Department of Epidemiology, Mailman School of Public Health, Columbia University, New York, New York, United States of America; 19 Department of Psychology, San Diego State University, San Diego, California, United States of America; 20 Department of Nutrition, Gillings School of Global Public Health, University of North Carolina, Chapel Hill, North Carolina, United States of America; 21 Department of Neurology, Vagelos College of Physicians and Surgeons, Columbia University, New York, New York, United States of America; 22 Department of Epidemiology & Population Health and Department of Pediatrics, Albert Einstein College of Medicine, New York, New York, United States of America; 23 Center for Occupational and Environmental Health and Department of Public Health Sciences, University of California, Davis, California, United States of America; 24 Department of Biostatistics, School of Public Health, University Alabama at Birmingham, Birmingham, Alabama, United States of America; 25 Department of Medicine, University of California, San Francisco, California, United States of America; 26 Department of Medicine, Feinberg School of Medicine, Northwestern University, Chicago, Illinois, United States of America; 27 Department of Environmental and Occupational Health Sciences, University of Washington, Seattle, Washington, United States of America; 28 Department of Preventive Medicine, Feinberg School of Medicine, Northwestern University, Chicago, Illinois, United States of America; 29 Department of Epidemiology, Gillings School of Global Public Health, University of North Carolina, Chapel Hill, North Carolina, United States of America; 30 Department of Epidemiology and Biostatistics, College of Public Health, Temple University, Philadelphia, Pennsylvania, United States of America; 31 Department of Medicine, University of Colorado Denver Anschutz Medical Campus, Denver, Colorado, United States of America; 32 Department of Public Health Sciences, Pennsylvania State University, State College, Pennsylvania, United States of America; 33 College for Public Health and Social Justice, Saint Louis University, Saint Louis, Missouri, United States of America; 34 Division of Geriatrics, Geffen School of Medicine at University of California, Los Angeles, California, United States of America; 35 Department of Epidemiology and Biostatistics, Dornsife School of Public Health, Drexel University, Philadelphia, Pennsylvania, United States of America; 36 Heersink School of Medicine, University of Alabama at Birmingham, Birmingham, Alabama, United States of America; 37 Department of Biostatistics and Epidemiology and Center for American Indian Health Research, University of Oklahoma Health Sciences Center, Oklahoma City, Oklahoma, United States of America; 38 Division of Rheumatology, Department of Medicine, National Jewish Health, Denver, Colorado, United States of America; 39 Institute of Transportation Studies and Department of City and Regional Planning, University of California, Berkeley, California, United States of America; 40 Department of Neurology, Evelyn F. McKnight Brain Institute, Miller School of Medicine, University of Miami, Miami, Florida, United States of America; 41 Department of Obstetrics & Gynecology, University of California, San Francisco, California, United States of America; 42 Department of Social Medicine, Population and Public Health, University of California, Riverside School of Medicine, Riverside, California, United States of America; 43 Section of Endocrinology, Diabetes, Nutrition, and Weight Management, Boston University Chobanian & Avedisian School of Medicine, Boston, Massachusetts, United States of America; 44 Research Informatics Services, National Jewish Health, Denver, Colorado, United States of America; Environmental Research Center (CRE), ALGERIA

## Abstract

Neighborhood factors, encompassing social, built, and natural environments, may explain geographic differences in the impact of COVID-19 pandemic on populations. Data from pre-existing national, population-based cohorts could be leveraged to better understand how pre-existing conditions (both individual and neighborhood) contribute to risk factor development and disease progression. We catalogued spatial and neighborhood data in the Collaborative Cohort of Cohorts for COVID-19 Research (C4R), comprising 14 diverse US cohorts (>50,000 participants). The C4R sample is generally spatially and socially representative of the overall nation, with C4R’s calculated spatial coverage representing 28% of US land area and 52% of the total US population. However, C4R (vs. non C4R) areas were more urban, wealthy, with more foreign-born residents, and less car-dependent with lower proportion employed and green. Twelve cohorts collected neighborhood characteristics – most commonly social environment data on neighborhood socioeconomic status– based on participants’ addresses. The most common built environment measures were related to food access, followed by other destination-based measures such as walkability. Natural environment data were available in the fewest cohorts, with emphasis on air quality or greenspace. This work provides clarity on available neighborhood and spatial data and facilitates future harmonization of data from C4R cohorts. Ultimately, this may enable future longitudinal and comparative analyses of neighborhood influences on COVID-19.

## Introduction

As the coronavirus-19 (COVID) pandemic unfolded across the US, the burden of COVID infection, severity, poor recovery, and mortality were disproportionately high in low-income and lower resourced communities [1–3]. Extant research on how environmental factors within communities were associated with COVID outcomes [4–7] demonstrated higher infection and mortality rates in larger metropolitan areas [8], higher severity of COVID infection with higher levels of air pollution [9–11], and reduced risk of COVID mortality with greater greenspace exposure [12–14]. Despite the importance of social, natural, and built environments as powerful determinants of health behaviors and conditions, prior efforts to disentangle the role of environments during the COVID pandemic were limited by ecologic designs that lacked individual longitudinal data. These data are necessary to disentangle existing health trajectories and health risks that affect COVID susceptibility and severity from direct environmental impacts on COVID infection, outcomes, or long-term prognosis.

Several national, population-based longitudinal studies measure complex risk factor development, progression of disease, and longitudinal, life-course predictors of health. Within the context of the COVID pandemic, these cohorts presented a unique opportunity to examine how pre-existing health conditions and personal characteristics contributed to COVID risks. They also allow the examination of potential long-term changes in behaviors or health stemming from the pandemic period (e.g., decreased or increased physical activity from lockdown). Established early in the pandemic, the Collaborative Cohort of Cohorts for Covid-19 Research (C4R) brings together 14 cohort studies (S1 Table, list in abbreviations) that have followed participants across the US for between 10 and 50 years. All cohorts are listed in abbreviations; details on each cohort and C4R published elsewhere [15]. Briefly, C4R has collected information on COVID vaccination, infection, recovery, and mortality, as well as behavior and psychosocial outcomes from over 50,000 participants combined.

Outside of the efforts of C4R, many of these cohorts have previously collected spatial and neighborhood data about their participants, often over many exams and decades. These deeply phenotyped, longitudinal, well-characterized cohorts span diverse populations, life phases, and geographies. Thus, they offer sufficient variability to better understand how environmental context impacts COVID and other health outcomes. Yet efforts to operationalize environmental data across different cohorts have been decentralized and are often led by independent investigators, resulting in variable data collection protocols and metrics [16–19]. Collaboration among cohorts and harmonization of environmental data encourages the identification of factors impacting COVID outcomes and disparities. Specifically, harmonized geospatial infrastructure could support future longitudinal and comparative analyses. The first step toward this harmonization is to identify and quantify which data exist, at what times, and for which cohorts.

This paper provides a comprehensive inventory of the available spatial data across all C4R cohorts and examines the spatial extent of C4R participants. We systematically assessed which data are available within each cohort and quantified the representativeness of these combined cohorts within the US. Our longer-term goal was to document available data and inform measure harmonization and development within C4R. We expect this work will facilitate well-powered research on the influence of spatial/neighborhood characteristics on COVID-19 outcomes and pandemic shifts across diverse geographies and participants.

## Methods

### Cohort geospatial inventory

In 2021, three study team members (JAH, LMB, MPJ) drafted a geospatial inventory form to capture essential geospatial methods and measures within each C4R cohort (S4 Appendix in S4 File). The form documents each cohort’s: (1) format and frequency of collecting and geocoding addresses, as well as the types of addresses collected (e.g., residential, work, school) and level of geocoding (e.g., latitude-longitude or Zip + 4); (2) types of neighborhoods assessed, such as administrative data appended to geocodes (e.g., city, state, county) and the availability of buffers (e.g., Euclidian/straight line or network/street buffers); and (3) measures of the environment collected, created, or attached to participant geocodes. Specifically, the form assessed available neighborhood-level measures (i.e., already linked to participant locations) of the natural environment (e.g., greenspace, air quality), built environment (e.g., food stores, social and walking destinations, housing density), and social or economic environment (e.g., racial and ethnic composition, neighborhood residents’ socioeconomic status). Within each of these three broad categories, the form asks about specific neighborhood metrics, the method of collection or derivation (e.g., survey, GIS, audit), the data source and year, the level of measurement (e.g., census tract or Euclidian buffer), and the time period(s) in which the measure was linked to locations (e.g., baseline, visit year). Lastly, the form collects COVID-specific neighborhood measures: number of cases, number of deaths, vaccination rates, policies, and disparity indices (e.g., race- or income-based differences). For each COVID-specific neighborhood metric, the form asks for the specific metrics available, the source of the data (e.g., survey, government agency), the level of measurement (e.g., city, zip), and the timing of measurement (e.g., by years, months). The draft inventory form was reviewed for clarity and completeness by three investigators and was piloted to assess usability in one of the C4R cohorts before finalization for use with the remaining 13 C4R cohorts.

### Cohort study design

The C4R Study design, as previously described [20], followed a cohort ancillary studies model. Researchers in each cohort study were directly responsible for accomplishing data collection in accordance with the standard protocols and under the supervision of their own observational studies monitoring board, steering committee, institutional review board (IRB), and any other applicable regulatory authorities. Columbia University served as the Data Coordination and Harmonization Center (DCHC) for C4R (Columbia University Institutional Review Board, IRB-AAAT3035). A full list of cohort IRBs supervising implementation of the C4R protocols is provided in S2 Table. For the purposes of the work presented in this manuscript, data were accessed at Drexel University from February 28, 2022, to April 26, 2024. Drexel IRB determined that work within this manuscript was not human subjects research. Cohort data inventories were conducted beginning February 28, 2022, and finalized January 9, 2023. Study data were previously recorded as part of the C4R Study and were handled in accordance with all applicable confidentiality and data protection regulations.

### Cohort geospatial data acquisition process and ensuring confidentiality

We (JAH, LMB, MPJ, STD, TC) contacted representatives from each of the 14 C4R cohorts requesting data manuals for each cohort’s geospatial data from which information was extracted using the geospatial inventory form. We also looked for published manuscripts using measures of interest. This was done to minimize effort for each individual cohort. When each inventory was complete, we confirmed with the cohort that our assessment was accurate.

To provide data on the geographic coverage of C4R, we requested a list of geospatial units for participants’ residential locations. We requested cohorts to provide the geographic administrative units to which participant addresses were geocoded (e.g., census tract, ZIP code, county, state) which were the smallest spatial resolution shareable without potential participant identification. While select cohorts have workplace locations, we did not include these. Geocoding data were provided to us in an anonymized format (i.e., as a list of administrative geographies) and no individual participant data were linked to these locations.

### Public Data Sources Linked to Geographic Coverage of C4R

Utilizing the American Community Survey (ACS) 2015−2019 5-Year estimates and the geospatial data acquired for cohorts, we derived relevant population, social, and economic context information for a comparative analysis between areas where C4R participants have lived and those in which C4R participants have not lived. We used census tracts that match the 2015−2019 ACS boundaries, derived from the National Historical Geographic Information System (NHGIS) 2019 Census Tract Boundary File: United States and Puerto Rico [21]. These measures included total population density (pop/km^2^), median household income, as well as percentages of older adults (≥65 years, ≥ 85 years), non-Hispanic Whites, foreign-born residents, home ownership (% rent, % own), residents below poverty, residents with formal educations (% with high school (HS) degree, % with college degree), residents employed, commuting by car, and crowding. Index of Concentration at the Extremes (ICE) was calculated from ACS data using standard methods [22] for both the high-income White households versus low-income Black households and the high-income White non-Hispanic households versus low-income Hispanic households. ICE is scaled from −1 to +1 where −1 indicates that 100% of the population in the given area is concentrated among the most deprived group, and +1 means that 100% of the population is concentrated in the most privileged group.

Data regarding urbanicity were derived from the McAlexander et al. community type classification [23]. We operationalized urbanicity as the percent of total tracts that were classified as “high density urban”, “low density urban”, and “small town/suburban”, which make up all categories other than “rural” (and any undesignated tracts) as classified by McAlexander et al. To measure hospital access, we used staffed hospital bed counts per county from the Area Health Resources Files 2018–2019 to create a number of beds per 100,000 population density measure [24]. Data on greenness was derived from the 2019 National Land Cover Database (NLCD) [25] by including six categories of land cover previously used to generate a greenness measure (proportion of each category) at the census tract level [14]. We reclassified the categories into one single “green” category and used zonal statistics to get the percent areal coverage per census tract.

We captured metrics of neighborhood COVID burden from the Johns Hopkins COVID-19 dashboard (cases per quarter) and the Centers for Disease Control (CDC) COVID Data Tracker (vaccination rates) [26,27]. We aggregated daily COVID-19 case counts per county from the Johns Hopkins COVID-19 Dashboard to quarterly counts then used population to create a quarterly cases per 100,000 population density measure. The CDC publishes data on COVID-19 vaccination rates per county, which we used to indicate completed primary series vaccination rates at the end of each quarter (Jan-Dec). We examined case rates from Q1 of 2020 until Q4 of 2021, and vaccination rates from Q1 of 2021 (when a vaccine was available to a majority of the population) to Q4 of 2021. Despite access to data for 2022, we did not examine these rates due to a lack of reporting that likely makes these data not representative of overall COVID levels.

### Analyses

Inventory data on neighborhood measures within each cohort were grouped and summarized based on overall availability and construct. For example, since many cohorts did not obtain neighborhood housing type measures (i.e., age of housing, mix of multi-family vs. single family), but more collected home ownership (i.e., % within neighborhood who own or rent), we combined these into a single category (i.e., “Home ownership/housing conditions”). Similarly, many cohorts measured greenspace through park data or through satellite images, making it harder to differentiate between greenness measures and park access (which are related), so these were combined into “Greenspace/Parks.” Within each category, measures were counted and summarized based as 0 measures, 1 measure, 2–5 measures, 6 or more measures. For example, for “Access to Food Stores/Food Environment,” a cohort might have data on densities of fast-food establishments, restaurants, supermarkets, convenience stores, wholesale markets, and fruit and vegetable stands, as well as ratios of types of stores (e.g., healthy to unhealthy sources). This would count as 6 + measures. Similarly, data were summarized as being derived from GIS (e.g., the densities in the prior example) or derived from surveys to participants (e.g., self-reported access to fruit and vegetables within their neighborhood). We did not receive information from the cohorts indicating that they have audited any environmental characteristics using in-person audit tools. Finally, cohorts either had measures from only one time point (e.g., baseline or during an ancillary study) or multiple time points longitudinally (e.g., continuously, at each exam); therefore, we summarized data within each category using that dichotomy. All neighborhood measures were visualized in a heatmap that could illustrate data timing (color family), number of measures (intensity of color), and type of measures (pattern). Cleaning and summarization of inventory data was done in Excel, and the heatmap was created in R (ggplot2 package, RStudio 2023.06.1 with Base R version 4.3.0).

We received participant locations from each cohort in different aggregated geographic administrative levels and vintages. For example, one cohort provided counties in which participants had lived in 2015, while another provided tracts from four separate decennial censuses, ranging from 1980 to 2010. To harmonize these disparate boundaries, we merged and dissolved all files into one dataset and then overlaid it with 2019 census tracts from NHGIS. Tracts with at least 5% of their area overlapped by C4R participant geographies were included as “C4R areas” in the final tract-level datasets for comparison. All counties that these tracts fell in were used in final county-level datasets. Remaining census tracts and boundaries in the contiguous US were considered “non-C4R areas.” Only the contiguous US was considered for any aspect of this study, meaning that “national” measures refer to data for that subset only. All spatial processing was performed in ArcGIS Pro 2.9 (ESRI, Redlands, CA).

All public data sources on social, economic, and other contexts were joined to either the county- or tract-level geographies on matching Federal Information Processing Standards (FIPS) codes. These were then aggregated nationally, for C4R coverage area and for non-C4R coverage area. To calculate representativeness and coverage, total population and land area measures were summed in the aggregation. All other ACS 2015–2019 measures were aggregated by means. Therefore, final aggregated measures are means of tract-level measures, not percentages or indices of that level. For example, the HS Degree (%) measure is the mean of all tract-level percentages of persons with at least a high school diploma, rather than the total percentage of persons with a high school diploma. We used this method of aggregation for all additional measures. We described means and standard deviations (SD) for population, sociodemographic, economic, and environmental context data for C4R locations, non-C4R locations, and nationally. We used t-tests to calculate p-values comparing the means of C4R locations to means of non-C4R locations. All aggregation and analyses were done in Python 3.9 (Wilmington, DE).

## Results

### Address and geolocation data

COPDGene was the only cohort that did not centralize recorded addresses on inception of the cohort (i.e., address information kept within the 21 sites), instead centralizing addresses during a subsequent exam year once informed consent language was updated. Most cohorts updated their participants’ address information each time an exam was performed, with some cohorts updating information more frequently. For example, REGARDS recorded an initial address at the beginning of the study, then asked participants to update their addresses annually and confirmed addresses every six months during a follow-up call.

While collection and maintenance of address data was (and still is) done by all 14 cohorts, fewer cohorts (n = 12) have processed these participants’ addresses to identify their geographic location (i.e., geolocation or geocoding to a latitude-longitude for linkage to other geographic layers). Twelve of the cohorts (ARIC, CARDIA, COPDGene, FHS, HCHS/SOL, JHS, MASALA, MESA, NOMAS, REGARDS, SHS, SPIROMICS) have derived geospatial measures for administrative boundaries (e.g., tracts, zip codes, or counties), and six cohorts (ARIC, CARDIA, HCHS/SOL, JHS, MESA, REGARDS) have derived measures for Euclidian or network buffers (e.g., 1 km around residence). Cohorts varied in use of Euclidean or Network buffers and size of buffers around residential addresses; some cohorts calculated buffer sizes using metric (1 km, 3 km, 5 km) and others used imperial (0.5 mi, 1 mi, 3 mi, 5 mi). Buffers used were also specific to certain GIS measures (e.g., some cohorts measured distance to nearest roadway, which is not a buffer, while others calculated densities of road network within a buffer around a participant’s residence).

### C4R geospatial coverage

**Fig 1:**
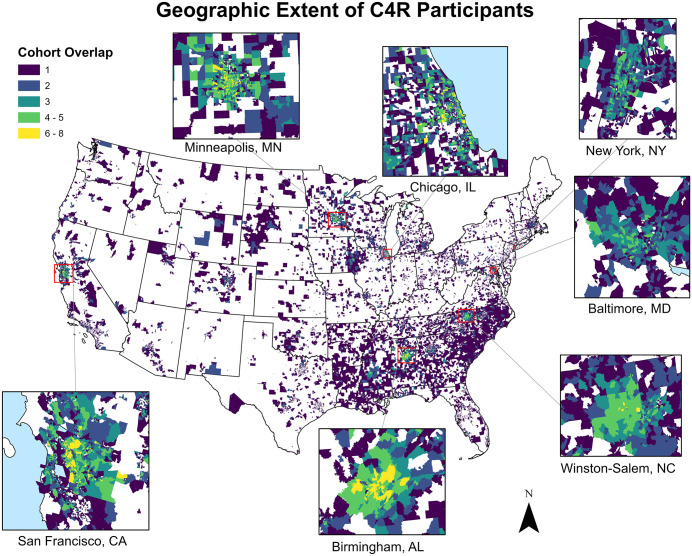
Geographic Extent of C4R Participants. Geographic coverage obtained from cohorts anonymized and aggregated by spatial unit (e.g., county, census tract) and time (e.g., baseline, most recent exam, all exams). Since participant locations were provided at varying time periods and geographies, the map does not necessarily represent locations during COVID-19 pandemic. Geographic boundaries shown in Fig 1 come from U.S. Census Bureau's TIGER/Line shapefiles.

C4R cohort participants covered 28% of the US contiguous land area, which includes 52% of the US total population (Table 1). Generally, compared to the US, C4R locations are more urban, with higher population densities, lower proportion non-Hispanic White residents, and higher foreign-born populations. The higher urbanicity of C4R locations can also be seen in higher proportions renting, a lower proportion commuting by car, and slightly lower greenness. Several locations within the US have participants from multiple C4R cohorts (“cohort overlap”) (**Fig 1**). These areas include San Francisco, CA, Minneapolis, MN, Chicago, IL, Birmingham, AL, Winston-Salem, NC, Baltimore, MD, and New York, NY. These cities represent potential opportunities to harmonize and attach raw environmental GIS data available in one cohort to another cohort that may not have these data.

**Table 1:** Population, sociodemographic, economic, and environmental characteristics for coverage of the C4R cohort geographies compared to national and areas not covered by C4R cohorts.

Neighborhood characteristic	C4R Locations^1^	National	Non-C4R Locations	p-value^7^
**Land Area (km**^**2**^)	2,153,275	7,654,980	5,501705	-----
**Total Population** ^**2**^	167,153,863	322,538,561	155,384,698	-----
**Urbanicity**^**3**^ **(%)**	82.69	75.61	68.72	-----
	Mean (SD)	Mean (SD)	Mean (SD)	
**Population density (pop/km**^**2**^)^**2**^	2613.08 (5687.63)	2087.58 (4598.84)	1575.32 (3114.43)	<0.001
**Age of residents: ≥ 65 (%)** ^**2**^	16.33 (8.28)	16.40 (8.14)	16.47 (8.0)	0.025
**Age of residents: ≥ 85 (%)** ^**2**^	2.08 (2.01)	2.06 (1.95)	2.04 (1.88)	0.003
**Non-Hispanic White Population (%)** ^**2**^	57.04 (30.52)	61.48 (29.91)	65.82 (28.64)	<0.001
**People of Color Population (%)** ^**2**^	42.96 (30.52)	38.52 (29.91)	34.18 (28.64)	<0.001
**Foreign-Born Residents (%)** ^**2**^	13.27 (13.86)	12.65 (13.59)	12.04 (13.29)	<0.001
**Median Household Income** ^**2**^	68662.23 (35392.07)	66977.19 (33467.29)	65321.33 (31372.80)	<0.001
**Rent (%)** ^**2**^	38.53 (23.51)	36.56 (22.95)	34.63 (22.22)	<0.001
**Own (%)** ^**2**^	61.47 (23.51)	63.44 (22.95)	65.37 (22.22)	<0.001
**Persons Below Poverty (%)** ^**2**^	14.85 (12.00)	14.68 (11.60)	14.51 (11.20)	<0.001
**HS Degree (%)** ^**2**^	87.88 (9.89)	87.32 (10.36)	86.76 (10.77)	<0.001
**College Degree (%)** ^**2**^	33.18 (20.32)	30.7 (19.35)	28.27 (18.01)	<0.001
**Employed (%)** ^**2**^	93.99 (4.68)	94.19 (4.42)	94.38 (4.14)	<0.001
**Commuting by Car (%)** ^**2**^	83.03 (17.57)	84.81 (15.57)	86.56 (13.10)	<0.001
**ICE (high-income White households vs. low-income Black households)** ^**4**^	.161 (.258)	.181 (.227)	.201 (.188)	<0.001
**ICE (high-income White non-Hispanic households vs. low-income Hispanic households)** ^**4**^	.201 (.197)	.196 (.194)	.192 (.193)	<0.001
**Crowding (occ housing units w/ > 1 person per room, %)** ^**2**^	3.50 (5.07)	3.54 (5.15)	3.58 (5.22)	0.035
**Hospital Beds/100k Population** ^**5**^	282.06 (396.79)	300.39 (493.41)	344.55 (669.18)	0.001
**Greenness (%)** ^**6**^	29.21 (26.71)	31.54 (27.85)	33.82 (28.74)	<0.001

1 Averages represent the areas where C4R cohort participants have recorded addresses. All geographies were aggregated across cohorts and available exam periods. Estimates represent the reaggregated population, sociodemographic, economic, and environmental characteristics for locations with participants and locations without participants allocated by geographic centroid.

2 Data from ACS 2014–2018

3 Urbanicity defined using McAlexander, T. P., Algur, Y., Schwartz, B. S., Rummo, P. E., Lee, D. C., Siegel, K. R., … & McClure, L. A. (2022). Categorizing community type for epidemiologic evaluation of community factors and chronic disease across the United States. Social sciences & humanities open, 5(1), 100250.

4 ICE = Index of Concentration at the Extremes from calculated using standard equations [22].

5 Data on Hospital Beds/100k population from the Area Health Resources Files 2018–2019 to create a number of beds per 100k population density measure [24]

6 Greenness data from 2019 NLCD [25]

7 P-value comparing means in C4R locations to means in non-C4R locations using t-test.

### Neighborhood measures

**Fig 2:**
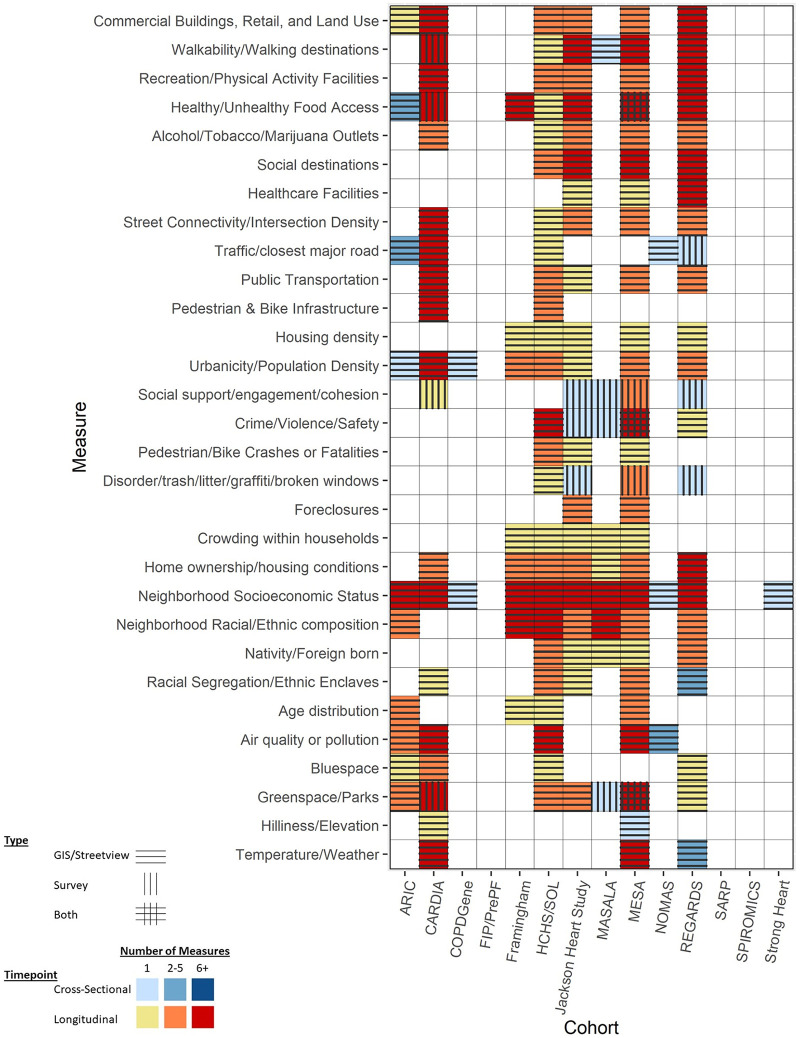
Neighborhood data in the C4R cohorts by timeframe, measure type, and number of measures. Abbreviations: ARIC, Atherosclerosis Risk in Communities; BCL, Biorepository and Central Laboratory; C4R, Collaborative Cohort of Cohorts for COVID-19 Research; CARDIA, Coronary Artery Risk Development in Young Adults; CONNECTS, Collaborating Network of Networks for Evaluating COVID-19 and Therapeutic Strategies; COPDGene, Genetic Epidemiology of COPD; FHS, Framingham Heart Study; HCHS/SOL, Hispanic Community Health Study/Study of Latinos; JHS, Jackson Heart Study; MASALA, Mediators of Atherosclerosis in South Asians Living in America; MESA, Multi-Ethnic Study of Atherosclerosis; NOMAS, Northern Manhattan Study; PASC, Post-Acute Sequelae of SARS-CoV-2 Infection; PrePF, Prevent Pulmonary Fibrosis; REGARDS, REasons for Geographic and Racial Differences in Stroke; SARP, Severe Asthma Research Program; SPIROMICS, Subpopulations and Intermediate Outcome Measures in COPD Study; SHS, Strong Heart Study.

In our inventory of the C4R cohorts’ geospatial data, twelve cohorts (ARIC, CARDIA, COPDGene, FHS, HCHS/SOL, JHS, MASALA, MESA, NOMAS, REGARDS, SHS, SPIROMICS) indicated that they have measured neighborhood geospatial data for their study participants (**Fig 2**). Two cohorts (FIP/PrePF, SARP) indicated they have not collected, processed, or linked any neighborhood geospatial data for their participants (blank columns, **Fig 2**).

### Built environment data

Built environment data were collected by nine cohorts (ARIC, CARDIA, FHS, HCHS/SOL, JHS, MASALA, MESA, NOMAS, REGARDS). The most common data collected by cohorts was access to food stores or food environment. These cohorts measured neighborhood amenities for retail food stores, such as wholesale clubs, grocery and convenience stores, chain stores, co-ops, fast-food, specialty, meat and fish, and supermarkets. Three cohorts (JHS, MESA, REGARDS) utilized data from the National Establishment Time Series (NETS) dataset to calculate changes in the business environments of their cohorts over time [28]. This dataset allows for cohorts to track the locations and types of businesses dating back to 1990, enabling longitudinal neighborhood change analysis. CARDIA, HCHS/SOL, JHS, MESA, and REGARDS all retained data on commercial buildings, land use, walkability, recreation/physical activity facilities, and alcohol/tobacco/marijuana outlets (counts per buffer or density per land area). These data were often derived from the NETS dataset, InfoUSA/ReferenceUSA, or similar dataset offerings from ESRI (i.e., ArcGIS Business Analyst). Traffic and road infrastructure, often primarily calculated using distance to arterial roads, were measured by eight cohorts (ARIC, CARDIA, HSCS/SOL, FHS, JHS, MESA, NOMAS, REGARDS); this was disparate from street connectivity, measured using both density of intersections, ratios of Network to Euclidean buffers, and other measures of connectivity.

### Social environment data

Social environment data were the most plentiful data type connected to the C4R cohorts. Most cohorts have collected aggregated data on neighborhood socioeconomic status and data on urbanicity and population density, with all cohorts in possession of neighborhood-level data reporting some level of socioeconomic status. Most commonly, cohorts linked participant data to neighborhood employment data, median household income, education levels of residents, and poverty derived from the US decennial census and ACS. Cohorts that measured urbanicity of their participants’ communities often relied on rural-urban commuting area (RUCA) codes, a tract-based classification that uses standard census measures of population density, levels of urbanization, and journey-to-work commuting to characterize all US census tracts with respect to their rural/urban status [29].

Five cohorts reported measuring levels of neighborhood social engagement and cohesion (CARDIA, JHS, HCHS/SOL, MASALA, MESA). As expected for these types of constructs, these measures were assessed using survey tools administered to cohort participants. Uniquely, MESA measured these constructs simultaneously at the community level through administration of the survey to nearby, non-MESA participants and then created neighborhood estimates within a set buffer of MESA participant residences using Bayesian estimation techniques [30]. Five cohorts incorporated levels of violence and crime (HCSC/SOL, JHS, MASALA, MESA, REGARDS). Sometimes perceptions of violence or crime were measured (e.g., in MESA and their community survey), while other times crime data were GIS-based from local municipalities or the CrimeRisk Index (provided through ESRI from Applied Geographic Solutions) [31].

### Natural environment data

Natural environment data were the least available construct of geospatial data available among cohorts. Nine measured air quality (ARIC, CARDIA, HCHS/SOL, FHS, JHS, MESA, NOMAS, REGARDS, SPIROMICS), five measured greenspace/parks (CARDIA, HCHS/SOL, JHS, MASALA, MESA, REGARDS), three measured bluespace (CARDIA, HCHS/SOL, REGARDS) and three measured temperature/weather (CARDIA, FHS, MESA), and only two measured hilliness/elevation (CARDIA, MESA). Cohorts that measured air quality included data on multiple pollutants, especially nitrogen dioxide, ozone, particulate matter less than 10 um or 2.5um (PM_10_, PM_2.5_), and nitrous oxide, measuring them in many different ways (e.g., modeling, averages during specific time periods). Some cohorts collected air quality data from EPA air pollution sensors [32], while many used more complex modeling techniques to estimate fine-scale air pollution [33]. Cohorts that measured greenspace/parks with park access generally quantified park density, size, and characteristics frequently sourced from local parks departments, city governments, or ParkServe [34]. When measuring natural vegetation, cohorts often used the National Land Cover Database from the USGS [25] or the Normalized Difference Vegetation Index (NDVI), a measure of vegetation calculated using ArcGIS from Landsat or Moderate Resolution Imaging Spectroradiometer (MODIS) satellite imagery [35]. Three cohorts measured some type of bluespace data (CARDIA, HCSC/SOL, REGARDS), measuring distances to the nearest shoreline and surface land use that is coded as water or ice. The three cohorts (CARDIA, FHS, MESA) that measured temperature (e.g., average variation, extreme temperature events) and weather (e.g., precipitation levels) tended to source data from the National Oceanic and Atmospheric Administration (NOAA) local climatological data archive [36] or local monitors (e.g., Logan International Airport). Almost no cohorts measured light pollution or noise, so these were not included in Fig 2.

### COVID Pandemic contextual data

To date, none of the surveyed cohorts have linked neighborhood-level COVID data to participants’ addresses. While the C4R project gathered self-reported COVID within participants’ friend and family networks, a large gap remains in connecting participants to the COVID levels or pandemic responses (e.g., policies) they experienced around their residences.

### Forthcoming geospatial or neighborhood data

On or before January 2024, several cohorts reported geospatial measures being developed by investigators (through funded projects, ancillary studies, or approved manuscript proposals). These measures do not appear in this manuscript as they are not yet readily available for outside researchers through the coordinating centers. Examples include new segregation measures in ARIC, new disaster and climate measures in MESA, and new segregation, redlining, and neighborhood disinvestment measures in JHS (**S3 Table**).

#### 3.8.1. Sociodemographic, economic, environmental, and COVID representativeness of C4R geographics.

Areas where C4R participants have lived or presently reside are more dense, urban, affluent, formally educated with a higher proportion foreign born residents and less car-dependent, with lower proportions employed and green than the non-C4R locations and the US overall (**Table 1**). Variability exists across cohorts (not shown). However, unsurprisingly, since many cohorts are based at institutions located in major cities, the geographic coverage of C4R is more dense (2613.08 population/km^2^) and urban (82.69) than the entire US (2087.58 and 75.61, respectively) and non-C4R locations (1575.32 and 68.72, respectively, p < 0.001). Relatedly, C4R areas have higher percentages of residents with a college degree and higher median household income, but also have a higher proportion of residents renting, below the poverty line, and unemployed. Tracking with their higher urbanicity, C4R locations have lower percentage of residents commuting by car and more racial and ethnic diversity as represented by both higher proportion foreign-born residents and lower non-Hispanic White residents. Interestingly, using ICE comparing only high-income White households to low-income Black households showed C4R locations have less concentrated privilege, while using ICE comparing high-income non-Hispanic White households to low-income Hispanic households showed C4R locations to have more concentrated privilege than non-C4R locations or the national average.

Estimates for the community COVID context of C4R participant locations showed that they generally experienced higher rates of infection and slightly higher rates of vaccination (**Table 2**). For example, in Q2 and Q3 of 2020, when the pandemic first peaked, the case rates for C4R locations were 536.74 cases per 100,000 and 1498.62 cases per 100,000 people, respectively, while the national rates were 489.68 cases per 100,000 people and 1435.37 cases per 100,000 people, respectively. We observed lower case rates in C4R locations in Q4 of 2020 and Q4 of 2021 than the case rates of the nation and non-C4R areas. Vaccination rates were always higher in the C4R locations than for the nation and non-C4R areas.

**Table 2:** COVID Experience for the C4R cohort geographies^2^.

	Case Rate (per 100,000)^1^		Vaccination Rate (%)^1^	
**Variable**	**C4R Locations** ^**2**^	**National**	**C4R Locations** ^**2**^	**National**
**2020**				
**Q1**	23.06	18.95	NA	NA
**Q2**	536.74	489.68	NA	NA
**Q3**	1498.62	1435.37	NA	NA
**Q4**	4501.01	4737.42	NA	NA
**2021**				
**Q1**	2887.01	2780.86	13.75	13.53
**Q2**	859.64	818.49	32.53	30.73
**Q3**	3600.22	3527.58	40.96	38.59
**Q4**	3432.20	3522.29	48.73	47.14

1 COVID-related variables were derived from the Johns Hopkins COVID-19 dashboard (cases per quarter) and the CDC COVID Data Tracker (vaccination rates) [26,27].

2 Numbers represent the COVID context and experience of areas where participants are located. They do not represent case rates or vaccination rates for C4R participants themselves. Averages represent the areas where C4R cohort participants have recorded addresses. All geographies were aggregated across cohorts and available exam periods. Estimates represent the areal-weighted, reaggregated characteristics for locations with participants and nationally.

## Discussion

In the current study, we cataloged the available geospatial and neighborhood data and geospatial coverage of the 14 cohorts included in C4R. Twelve of the 14 cohorts had at least one neighborhood measure available at the time of this inventory. Social environmental data were the most common data element, with most cohorts having data on at least one neighborhood measure of socioeconomic status. The most common built environment measures were related to food access, followed by other destination-based measures such as walkability. Natural environment data were available in the fewest cohorts, with emphasis on air quality or greenspace. The C4R sample is generally spatially and socially representative of the overall nation, covering 28% of US land area and 52% of the US population. Areas where C4R participants have lived or currently reside differed from non-C4R locations and the nation overall, although these differences were modest.

### Application of findings to research questions

Our inventory provides a comprehensive overview of environmental data available across C4R cohorts and highlights opportunities for future harmonization relevant to COVID research. The comprehensive characterization of pre- and mid-pandemic neighborhood conditions with sufficient geospatial variability and participant diversity can enable interdisciplinary research. Specifically, these pre- and mid-pandemic data allow the examination of environmental factors’ contribution to COVID risk and disparities, including on infection, morbidity, mortality, persistent symptoms, and vaccination uptake. Place-based differences in COVID impact by urbanization or city characteristics have been demonstrated across countries [37,38] and within the US [39]. Examinations of how neighborhoods impacted COVID outcomes informed previous prevention efforts, such as opening up park space or closing streets to create protected pedestrian spaces [40,41]. This work can also inform the creation of environments that are resilient to future public health disasters [39,42,43]. The geographic representation and sociodemographic diversity of the C4R cohorts, along with their combined sample size, permits research into how policies or neighborhoods contribute to COVID and related health disparities. Emergency measures in other domains implemented by some locales, such as eviction moratoriums, could be helpful to public health and used as a basis for future policies to protect populations [39,44–47].

The geographic and individual diversity of C4R could also be leveraged to understand how neighborhoods and policies differentially impact COVID and other health outcomes by participant characteristics such as race and ethnicity, sex/gender, age, and socioeconomic status. This may help identify the conditions and populations for which policies are most effective. Ultimately, while the COVID pandemic intensity wanes, lessons learned about neighborhoods during the COVID pandemic and recovery period can be applied to enhance future disaster preparedness [48]. However, this can only happen if efforts are made to integrate individual-level risk information to avoid both ecological bias [48] and confounding by prior conditions. Longitudinal data from C4R helps to overcome some of these limitations, allowing for better control of changing ecological conditions over time.

C4R cohorts’ neighborhood environment and contextual data could also be leveraged to understand critical shifts that occurred in built, social, and natural environments *post*-pandemic and their subsequent health impacts. These may be primary shifts that occurred early during the pandemic (e.g., policies around eviction, stay-home orders, pedestrianized streets) or enduring changes to retail environments, physical spaces, economic conditions, or population distributions. Evidence suggests that early pandemic prevention measures lowered pollution levels, limited access to public spaces, closed retail establishments, and produced lost income [49–51]. A number of cities made intentional health-supportive changes to their environments (e.g., pedestrianized streets, parklets), with varying success retaining these as the pandemic has progressed [52–54]. Similar evidence has begun to emerge regarding shifts in health-related contexts and social environments, including crime [55–57], neighborhood trust or social cohesion [58,59], and movement patterns [60–62]. As the country and world ease into a “new normal,” it is essential to understand long-term impacts of the pandemic period on our lived experience, including neighborhood conditions. Further, the addition of novel forthcoming geospatial measures (e.g., segregation, resilience to climate disasters) could be integrated into existing datasets to encourage additional inquiries as conditions evolve. Connecting C4R neighborhood data to the extensive pre-pandemic (1975–2020) and planned (2020-present) deep phenotyping in the cohorts on physical function, cardiac measures, brain measures, sleep, biomarkers, and genotyping, among others, overcomes confounding by lack of knowledge regarding previous health behaviors and trajectories. Linking neighborhood data with these cohorts would provide an opportunity to understand how the COVID pandemic and related neighborhood or community changes affected other health conditions commonly assessed across the cohorts, such as cardiovascular risk, aging, and related diseases.

### Future harmonization

Despite the myriad opportunities afforded by C4R neighborhood data, challenges remain for harmonization and future work. First, the pandemic disrupted data collection and infrastructure systems across the country and world. Specific to the US, there are known concerns about the quality and viability of Census 2020 and American Community Survey data that include the year 2020 (i.e., all five-year estimates from 2016–2025). This is similar for any other measure traditionally collected and processed in-person (e.g., building inspection measures), but is less likely to impact remote sensing measures (e.g., greenness). Furthermore, the validity and meaning of these measures may have changed due to pandemic restrictions (e.g., land uses designated for commercial purposes were closed and their public functions suspended). Second, although many cohorts have measures within a domain, these measures use different metrics, were collected at different levels of resolution (e.g., counties vs. census tracts), or were based on different underlying administrative and feature data. For example, some cohorts may source land use measures from parcel data collected in specific jurisdictions while others use national satellite imagery. Similarly, some may calculate intersection density for street networks while others use block length or a ratio of Network buffers to Euclidean buffers. Existing survey data are rich and comprehensive within each cohort but very sparse across cohorts, and without it, we may lack the ability to understand individuals’ perceptions of neighborhood amenities or culture pre-, mid-, and “post”-pandemic. Even within cohorts that collect survey data on perceived safety, social cohesion, and belonging, there are no signs of convergence toward a single standard set of tools. These inconsistencies make harmonization of existing data challenging. In addition, in several cohorts, neighborhood and spatial data were created, stored, and managed by ancillary studies rather than centrally within the coordinating center that currently interfaces with C4R. Harmonization is further hindered by the sensitive nature of spatial data; there are human subjects concerns when sharing geospatial data such as addresses and the various cohorts and university IRBs have different restrictions about the identifiability of participant latitudes-longitudes.

Despite privacy concerns, options for harmonization exist that circumvent some of these obstacles. One option is to create national-level metrics at a small administrative scale (i.e., block groups or tracts) that could be linked by each cohort to their participant locations. Alternatively, the creation of a national set of gridded metrics (i.e., as GIS rasters) would facilitate linkage to locations across a smooth, interpolated surface. These options create a balance between consistency across cohorts and ensuring data are backwards compatible, especially for measures where there is little to no ability to create historic metrics. While these workarounds may help with privacy concerns, some cohorts have limited internal resources, experience, and trained staff for managing geographic or spatial data to do the final linkage. Although harmonization is important to study neighborhood impact on COVID, pandemic impacts on everyday contexts, and their subsequent effects on health, these are only one arm of the many pressing scientific lines of inquiry possible for these cohorts. Ultimately, all cohorts are balancing different competing priorities and must remain accountable to their primary missions (e.g., cardiovascular, pulmonary), existing ancillary studies, and participants. Harmonization will require considerable financial resources across an extended timeline, and execution by a committed and experienced team of diverse researchers.

### Limitations and strengths

This paper provides a comprehensive description of the neighborhood and spatial data across cohorts but is not without limitations. First, many cohorts were missing some of the details on software for geocoding and, as in many transdisciplinary studies, we needed to establish common language around geography terminology and concepts (e.g., administrative boundary, uncertain geographic context problem, modifiable areal unit problem). Missing technical details may have occurred when neighborhood measures were compiled for ancillary studies but not distributed through the broader network of cohort researchers. When inventorying neighborhood data, the use of a survey and self-report by staff within cohort coordinating centers may introduce recall bias. We minimized these issues by collecting data documentation to confirm reported survey data and looking for published manuscripts using measures of interest. To facilitate harmonization, future work should prioritize documentation of software details and centralization of ancillary study datasets, code, and documentation at coordinating centers. In addition, as stated in our results, the inventory is a snapshot of what cohorts had in February-November 2022 and does not represent projects underway. For example, some cohorts had recently funded grants to incorporate new measures that were not yet calculated at the time of this inventory. We have tried our best to capture these forthcoming measures in the Supplemental files but may have missed upcoming research.

Our calculation of spatial extent has limitations which may introduce ecological bias and related issues, including modifiable area unit problem. Due to differences in human subject restrictions or levels of geocoding, cohorts sent residential location data at different spatial scales (e.g., cities, counties, census tracts) and across different spatial vintages (e.g., census 2000 tracts, census 2010 tracts). For anonymity, we only present data aggregated across all cohorts (i.e., counts of cohorts in a location, not the named cohort) and some cohorts additionally aggregated their data across time, providing all locations that a participant has lived across any exam. Additionally, cohorts with older adults (e.g., ARIC, MESA) have some participants who live in the southern US during the wintertime, and those wintertime locations are not necessarily captured in our presented data. Therefore, the data we presented on spatial representation combines time, spatial unit, and cohort. As such, the map of the geographic extent of C4R participants does not necessarily represent participants’ locations during the COVID-19 pandemic and the comparison of this extent to national demographics may include some spatial and/or temporal misclassification that could introduce bias. For research on specific exposure-outcome pairs, future work should try to gain approval and access to participant data including their COVID-19 specific outcomes and data collected at a spatial and temporal resolution best fitting the study’s specific aims, which would help to minimize ecological bias (e.g., modifiable area unit problem, ecological fallacy). The map also represents residential areas of C4R without weighting to account for differential counts of participants for given geographies (e.g., some census tracts may have one participant, some may have many). Similarly, our calculations of sociodemographic and COVID measures for the geographic extent required reaggregation of the spatial scale at which those data were provided (e.g., census tract for census, county for COVID data). This assumes an even distribution of each characteristic across the areal unit. Furthermore, we were limited to counties for national COVID data, and different reporting issues occurred at different points in the pandemic (lack of access to testing early in the pandemic, use of at-home tests later in the pandemic that may have not been reported to the city, etc.). Finally, to ease interpretation, all data presented are summaries and do not represent harmonized measures across the cohorts. More specific details on the availability of geospatial data for individual cohorts should be requested from the authors or directly from each cohort; access to these data should be requested through the approved processes of each cohort.

Despite these limitations, several strengths enhance the utility of this paper to guide future research on how environmental context shapes health outcomes and disparities. First, we used a centralized, systematic method for cataloging available geospatial data through a uniform data collection tool and process across all cohorts. Our graphical display of these data allows researchers to see potential synergies that would otherwise be difficult (or impossible) to recognize. In addition to sparking novel research questions and enabling well-powered analyses across diverse samples and regions, our clear summarization could be used as a model for other projects. Our method could be applied to those seeking to harmonize data across cohorts or as a guide for the creation of new measures (e.g., noise) that could be developed for one or more cohorts. Second, capturing key elements across these metrics, including differences in how data were collected (e.g., survey data vs. GIS data), allows researchers interested in utilizing these data to be informed about the inconsistencies across cohorts. This points researchers toward potential limitations of their analyses and provides information to interpret disparate study results across cohorts. Third, our systematic approach provides a roadmap for researchers who would like to collect their own geospatial data. For example, our collection process has already led to discussions across cohorts regarding best measures for specific environmental features. We believe that this summarization will encourage selection and operationalization of measures and methods that increase the coherence of the evidence created. This would enable even more scientific rigor and lead to the creation of larger and more diverse datasets that can answer highly complex questions about the intersection of environment and human health. Finally, our diverse and interdisciplinary team of national leaders in neighborhood health demonstrates best practices for team science. Our process ensured that multiple viewpoints were represented, ultimately encouraging shared methods and promoting collaborations with a wide range of disciplines with great potential to improve public health.

## Conclusion

In this study, we cataloged the geographic and spatial data available across a large geographically, socioeconomically, racially and ethnically diverse study of 14 pooled cohorts in the US. Our analyses described the spatial extent of the C4R population and examined representativeness compared to areas without C4R participants and compared to the nation overall, finding that C4R had relatively representative coverage of urban areas nationwide. We found that social environmental data were the most common types of contextual data collected, followed by built environment features. Our inventory also indicated that all cohorts were missing neighborhood COVID contextual data, several cohorts were lacking any geocoding or environmental data, and that even cohorts with contextual data had gaps in natural environment metrics, such as weather or heat, which may be relevant in an age of increasing climate-related events and vector-borne diseases [63–66]. Despite numerous advances in the assessment of neighborhood effects on health, our inventory of neighborhood measures highlights the need to develop a systematic methodology to facilitate harmonization. Agreement on common metrics, repositories of national environmental measures, and guidance documents for cohorts would encourage and enable valid and rigorous testing of the health effects of neighborhoods or their subsequent impacts on health disparities. Given the large quantity of data within the C4R project, methods will also need to be developed to integrate data across multiple spatial and temporal scales. Once harmonized, the associated data and methods could be shared widely to facilitate international collaborations and pooling of data for large-scale research projects (e.g., with the International Human Exposome Network (IHEN)). Work remains to understand the implications of the COVID pandemic on our neighborhoods, the way we interact with them, and their subsequent impact on our health and well-being. During a time when the environmental context is rapidly changing due to human activities and substantial climate change shifts, lessons learned from the pandemic period may help us create more resilient, health-supportive, equitable communities. This project opens possibilities for research collaborations to understand the health effects of neighborhood changes experienced during the pandemic and work toward sustainable, thoughtful neighborhood interventions to support health and well-being as future disasters arise.

## Supporting information

S1 Table Basic Characteristics of the 14 C4R cohorts March 1, 2020. (DOCX)

S2 Table Cohort Institutional Review Boards (IRBs) supervising implementation of the C4R protocols. Columbia University serves as the data coordinating center for C4R (IRB-AAAT3035). The study received initial IRB approval on December 4, 2020, with approval continuing through the current date (August 8, 2025). To date, four approval letters have been issued by the Columbia University IRB; these are provided in Supporting Document S5. This file is a list of site-specific IRB approval numbers for each study for the duration of the study period.
(DOCX)

S3 Table Forthcoming data not inventoried in this paper. We surveyed the cohorts’ coordinating centers on or before November 2022. Several cohorts reported geospatial measures being developed by investigators (through funded projects, ancillary studies, or approved manuscript proposals). These measures do not appear in this manuscript as they are not yet readily available for outside researchers through the coordinating centers.
(DOCX)

S4 File Appendix of the Survey to Inventory Geospatial and Neighborhood Data in C4R. This form was used by our team during data collection from cohort coordinating centers and administrators.
(DOCX)
