# Supplementary material for: Spatial and neighborhood data in the collaborative cohort of cohorts for COVID-19 Research (C4R)
Source: PLoS One. 2026 Jul 22;21(7):e0352170. doi: 10.1371/journal.pone.0352170 (PMC13390819; doi:10.1371/journal.pone.0352170)
Supplement: S1 Table — (DOCX) [file pone.0352170.s001.docx]

**S1 Table. Basic Characteristics of the 14 C4R cohorts March 1, 2020.**

| **Cohort** | **No. of Participants** | **Age Range, Years** | **Coordinating or Administrative Center Location** | **Field Sites/Recruitment Geographies** | **White** | **Black** | **Hispanic/Latinx** | **Asian American** | **American Indian** |
| --- | --- | --- | --- | --- | --- | --- | --- | --- | --- |
| ARIC | 6,690 | 75–97 | University of North Caroline at Chapel Hill (Chapel Hill, NC) | Three Field Sites:  Forsyth County, NC  Jackson, MS  Minneapolis, MN  Washington County, MD | ⚫ | ⚫ |  |  |  |
| CARDIA | 4,590 | 53–66 | University of Alabama at Birmingham (Birmingham, AL) | Four Field Sites:  Birmingham, AL  Chicago, IL  Minneapolis, MN  Oakland, CA | ⚫ | ⚫ |  |  |  |
| COPDGene | 7,731 | 50–90 | National Jewish Health (Denver, CO) | Clinical Centers:  Atlanta, GA  Ann Arbor, MI  Baltimore, MD  Birmingham, AL  Boston, MA  Denver, CO  Durham, NC  Houston, TX  Iowa City, IA  Los Angeles, CA  Minneapolis, MN  New York, NY  Philadelphia, PA  Pittsburgh, PA  San Antonio, TX  San Diego, CA  St. Paul, MN  Worchester, MA | ⚫ | ⚫ |  |  |  |
| FHS | 7,339 | 26–108 | Boston University (Boston, MA) | Framingham, MA | ⚫ | ⚫ | ⚫ |  |  |
| HCHS/SOL | 13,142 | 30–87 | University of North Caroline at Chapel Hill (Chapel Hill, NC) | Chicago, IL  Miami, FL  New York, NY (Bronx)  San Diego, CA |  |  | ⚫ |  |  |
| JHS | 5,306 | 38–102 | University of Mississippi Medical Center (Jackson, MS) | Jackson, MS |  | ⚫ |  |  |  |
| MASALA | 1,132 | 40–94 | University of California San Francisco (San Francisco, CA) | Greater Chicago area, IL  San Francisco Bay Area, CA |  |  |  | ⚫ |  |
| MESA | 4,683 | 65–103 | University of Washington (Seattle, WA) | Six Field Sites:  Baltimore, MD  Chicago, IL  Forsyth County, NC  Los Angeles, CA  Minneapolis, MN  New York, NY | ⚫ | ⚫ | ⚫ | ⚫ |  |
| NOMAS | 1,256 | 62–106 | University of Miami (Miami, FL)  Columbia University (New York, NY) | Northern Manhattan, NY | ⚫ | ⚫ | ⚫ |  | ⚫ |
| PrePF | 5,000 | 40–80 | University of Colorado (Denver, CO) | National mailings | ⚫ | ⚫ | ⚫ |  |  |
| REGARDS | 12,766 | 57–105 | University of Alabama at Birmingham (Birmingham, AL) | National:  Emphasis on Southeastern US | ⚫ | ⚫ |  |  |  |
| SARP | 397 | 18–80 | Penn State University College of Medicine, Hershey, PA | Atlanta, GA;  Boston, MA;  Charlottesville, VA;  Cleveland, OH;  Madison, WI;  Pittsburgh, PA;  Richmond, VA;  San Francisco, CA;  St Louis, MO;  Winston-Salem/Forsyth;  County, NC | ⚫ | ⚫ |  |  |  |
| SPIROMICS | 2,273 | 47–87 | University of North Caroline at Chapel Hill (Chapel Hill, NC) | Clinical Centers:  Ann Arbor, MI;  Baltimore, MD;  Birmingham, AL;  Chicago, IL;  Denver, CO;  Iowa City, IA;  Los Angeles, CA;  New York City, NY;  Philadelphia, PA;  Salt Lake City, UT  San Francisco, CA;  Winston-Salem, NC | ⚫ | ⚫ | ⚫ |  |  |
| SHS | 2,915 | 31–105 | University of Oklahoma (Oklahoma City, OK) | 13 American Indian tribes and communities in three geographic areas:  Near Phoenix, AZ;  Southwestern Oklahoma, OK;  Western & Central North (ND) and South Dakota (SD) |  |  |  |  | ⚫ |

**Abbreviations**: ARIC, Atherosclerosis Risk in Communities; CARDIA, Coronary Artery Risk Development in Young Adults; COPD, chronic obstructive pulmonary disease; COPDGene, Genetic Epidemiology of COPD; COVID-19, coronavirus disease 2019; C4R, Collaborative Cohort of Cohorts for COVID-19 Research; FHS, Framingham Heart Study; HCHS/SOL, Hispanic Community Health Study/Study of Latinos; JHS, Jackson Heart Study; MASALA, Mediators of Atherosclerosis in South Asians Living in America; MESA, Multi-Ethnic Study of Atherosclerosis; NOMAS, Northern Manhattan Study; PrePF, Prevent Pulmonary Fibrosis; REGARDS, REasons for Geographic and Racial Differences in Stroke; SARP, Severe Asthma Research Program; SHS, Strong Heart Study; SPIROMICS, Subpopulations and Intermediate Outcome Measures in COPD Study.
