# Supplementary material for: Spatial and neighborhood data in the collaborative cohort of cohorts for COVID-19 Research (C4R)
Source: PLoS One. 2026 Jul 22;21(7):e0352170. doi: 10.1371/journal.pone.0352170 (PMC13390819; doi:10.1371/journal.pone.0352170)
Supplement: S2 Table — Columbia University serves as the data coordinating center for C4R (IRB-AAAT3035). The study received initial IRB approval on December 4, 2020, with approval continuing through the current date (August 8, 2025). To date, four approval letters have been issued by the Columbia University IRB; these are provided in Supporting Document S5. This file is a list of site-specific IRB approval numbers for each study for the duration of the study period. (DOCX) [file pone.0352170.s002.docx]

**S2 Table. Cohort Institutional Review Boards (IRBs) supervising implementation of the C4R protocols.** Columbia University serves as the data coordinating center for C4R (IRB-AAAT3035). The study received initial IRB approval on December 4, 2020, with approval continuing through the current date (August 8, 2025). To date, four approval letters have been issued by the Columbia University IRB; these are provided in Supporting Document S5. Below is a list of site-specific IRB approval numbers for each study for the duration of the study period.

| **Cohort** | **Institutional Review Boards** |
| --- | --- |
| ARIC | University of North Carolina (11-0734) |
| CARDIA | University of Alabama at Birmingham (300006514, 300006580) |
| COPDGene | National Jewish (HS-1883a), Partners Human Research Committee (2007-P-000554/2; BWH); Baylor (H-22202), Columbia University (AAAC9324), Duke University Health System Institutional Review Board for Clinical Investigations (Pro00004464), Johns Hopkins Medicine (NA_00011524 / IRB00156198/CIR00058056), The John F. Wolf, MD Human Subjects Committee of Harbor-UCLA Medical Center (12756), Morehouse School of Medicine (07-1029), Temple University Office for Human Subjects Protections (11369), The University of Alabama at Birmingham (FO70712014), University of California, San Diego Human Research Protections Program (070876, 140070), The University of Iowa Human Subjects Office (200710717), VA Ann Arbor Healthcare System (PCC 2008-110732), University of Minnesota Research Subjects’ Protection Programs (0801M24949), University of Pittsburgh (PRO07120059), UT Health Science Center San Antonio (HSC20070644H), Health Partners Research Foundation (A07-127, PR2007P000554), University of Michigan Medical School Institutional Review Board (HUM00014973, HUM00140035), Minneapolis VAMC (4128-A), Institutional Review Board/Research Review Committee Saint Vincent Hospital – Fallon Clinic – Fallon Community Health Plan (1143), Reliant Medical Group (2592) |
| FHS | Boston University (H-41027) |
| HCHS/SOL | University of North Carolina at Chapel Hill (21-0732 – 324542, 335072, 356212, 391442; 07-1003 – 287027, 330589, 352145, 395850) |
| JHS | University of Mississippi Medical Center (1998-6004) |
| MASALA | Northwestern University (STU00019837-MOD0089), University of California San Francisco (20-33084) |
| MESA | University of Washington (STUDY00009029) |
| NOMAS | Columbia University (AAAA5489) |
| PrePF | Colorado Multiple Institutional Review Board, CB F490 (20-3101 / PAM006-1, PAM001-1, APP001-1, PAM004-2, PAM007-1) |
| REGARDS | The University of Alabama at Birmingham (020925004) |
| SARP | Wake Forest School of Medicine (IRB00066576) |
| SPIROMICS | Columbia University (AAAE9315, AAAT3035), The University of Iowa Human Subjects Office (202204389), Johns Hopkins Medicine Office of Human Research (NA_00035701 / CIR00091410; NA_00035701/CIR00066902), University of Michigan Medical School (HUM00193469 - Ame00140319, CR00092945), National Jewish Health (HS-2678-528), Temple University (21416), University of Alabama at Birmingham (120906004), University of California Los Angeles (18-000403-AM00019), University of California San Francisco (10-03169), University of Illinois at Chicago (2013-0939), University of North Carolina at Chapel Hill (10-0048 – 419905), University of Utah (00142457), Wake Forest University Health Sciences (00048727) |
| SHS | The University of Oklahoma (12902), Great Plains (14-R-07GP) |

ARIC = Atherosclerosis Risk in Communities Study; C4R = Collaborative Cohort of Cohorts for COVID-19 Research; CARDIA = Coronary Artery Risk Development in Young Adults; COPDGene= Genetic Epidemiology of COPD; FHS = Framingham Heart Study; HCHS/SOL = Hispanic Community Health Study/Study of Latinos; JHS = Jackson Heart Study; MASALA = Mediators of Atherosclerosis in South Asians Living in America; MESA = Multi-Ethnic Study of Atherosclerosis; NOMAS = Northern Manhattan Study; PrePF = Prevent Pulmonary Fibrosis; REGARDS = REeasons for Geographic and Racial Differences in Stroke; SARP = Severe Asthma Research Program; SPIROMICS = Subpopulations and Intermediate Outcome Measures in COPD Study; SHS = Strong Heart Study
