# Supplementary material for: Spatial and neighborhood data in the collaborative cohort of cohorts for COVID-19 Research (C4R)
Source: PLoS One. 2026 Jul 22;21(7):e0352170. doi: 10.1371/journal.pone.0352170 (PMC13390819; doi:10.1371/journal.pone.0352170)
Supplement: S3 Table — We surveyed the cohorts’ coordinating centers on or before November 2022. Several cohorts reported geospatial measures being developed by investigators (through funded projects, ancillary studies, or approved manuscript proposals). These measures do not appear in this manuscript as they are not yet readily available for outside researchers through the coordinating centers. (DOCX) [file pone.0352170.s003.docx]

**S3 Table. Forthcoming data not inventoried in this paper.**

We surveyed the cohorts’ coordinating centers on or before November 2022. Several cohorts reported geospatial measures being developed by investigators (through funded projects, ancillary studies, or approved manuscript proposals). These measures do not appear in this manuscript as they are not yet readily available for outside researchers through the coordinating centers.

| **Cohort** | **Forthcoming Data** | **Comments** |
| --- | --- | --- |
| ARIC | Racial segregation/ethnic enclaves; Age distribution; Nativity/Foreign Born; Home ownership/housing conditions; Neighborhood Racial/Ethnic composition; Greenspace/parks; Roadway distances and densities; Urbanicity; Gentrification; Healthcare resources. | Funding provided through the ARIC Neurocognitive Study (Whitsel, Stewart, Kucharska-Newton) |
| CARDIA | Additional measures through Y35 (all measures noted)  Field audits, school quality, neighborhood SES measures, urbanization, crime, climate, land cover, pollution.  Discriminatory mortgage lending measures  Area-level measures related to stigma and resilience-promoting in sexual and gender minority individuals. | New measures on discriminatory mortgage lending grant (5R01HL155187, PI Richardson), sleep study (5R01HL152442, PIs Carnethon and Knutson), and CHAMBERS grant (5R01HL149866, PI Beach) |
| COPDGene | PM2.5 and Ozone values in progress under ancillary study  ADI and SVI | Funding provided through Environmental Factors Predicting Risk of Severe COVID Infection (NIH R21 ES032973, Regan) |
| FHS | None noted. | ----- |
| HCHS/SOL | Neighborhood Greenness; Walkability.  Air Pollution.  Home, Worksite, Neighborhood and Transportation Environment Exposures. | Funding provided by “Neighborhood Greenness and Cardiometabolic Health among Hispanics in the HCHS/SOL Study”: SOL-Greenness Study, (NHLBI 1R01HL148880, MPIs: Brown & Szapocznik)  Funding provided by “SOLAir: Environmental Factors and Diabetes Development in Latinos”: SOL-Air Study (NIEHS 5R01ES030994, PI: Kaufman)  Funding provided by “Characterization of Sedentary Patterns and Cardiovascular Disease Risk Markers in Hispanics/Latinos” (NHLBI 5R01HL148463, PI: Carlson) |
| JHS | Racial segregation; household poverty neighborhood change; access to healthy and unhealthy food stores; land use physical/built environment; social environment; healthcare facility access.  Google streetview imagery virtual audits. | Funding provided through Cardiometabolic Risk Development and Management in Changing Neighborhoods: the Jackson Heart  Study (R01HL148431, PI: Barber) |
| MASALA | None noted. | ----- |
| MESA | Additional measures through Exam 7 (all measures noted)  Climate-related disaster; supports for healthy aging; Noise | Funding provided by NIH-NIA“Contribution of Longitudinal Neighborhood Determinants to Cognitive Health and Dementia Disparities within a Multi-Ethnic Cohort”: MESA Neighborhood and Aging Study (R01AG072634, PI: Hirsch) |
| NOMAS | Neighborhood Greenness; Neighborhood Income; Walkability; Crime; Food environment; Roadway distances and densities | Funding provided by “Greenness, Cognitive Performance and Vascular Outcomes in the NOMAS Study”: NOMAS-Greenness Study (NIA 1RF1AG074306, MPIs: Brown/Rundek/Szapocznik) |
| PrePF | None noted. | ----- |
| REGARDS | Climate/disaster; Noise, structural racism, neighborhood segregation, systemic racism | Funding provided by NIH RF1NS127606 Colabianchi, 1R01HL164116 Dean, R01CA263770 Akinyemiju, AHA grant Kamin-Mukaz) |
| SARP | None noted. | ----- |
| SPIROMICS | None noted. | ----- |
| SHS | None noted. | ----- |
