# Supplementary material for: Spatial and neighborhood data in the collaborative cohort of cohorts for COVID-19 Research (C4R)
Source: PLoS One. 2026 Jul 22;21(7):e0352170. doi: 10.1371/journal.pone.0352170 (PMC13390819; doi:10.1371/journal.pone.0352170)
Supplement: S4 File — This form was used by our team during data collection from cohort coordinating centers and administrators. (DOCX) [file pone.0352170.s004.docx]

**S4 Appendix**

**Inventory of Geospatial and Neighborhood Data in C4R**

Thank you for your ongoing support, commitment, and work within the Collaborative Cohort of Cohorts (C4R) project! As part of an ongoing effort to inventory, and eventually harmonize, data across cohorts we are collecting information about geospatial and neighborhood data within your cohort.

This form will ask questions about participant location data, geocoding/geolocating, and environmental variables (including natural, built, social, and covid data). Please try your best to answer information accurately for your cohort. The form serves as a template; we welcome reporting on additional variables unique to your cohort. We are also requesting any codebooks, data documentation, forms, or data dictionaries that you may feel are useful for this project.

**Respondent Information:**

Please provide the following to facilitate follow-up and clarification. Your information will not be shared and will only be used to contact you regarding the C4R geospatial/neighborhood data.

1. First Name: __________________________________________________________________________________
2. Last Name: __________________________________________________________________________________
3. Email: __________________________________________________________________________________
4. Phone: __________________________________________________________________________________
5. Institution: __________________________________________________________________________________
6. Cohort: __________________________________________________________________________________
7. Role within the Cohort: __________________________________________________________________________________
8. Date completed or interviewed (date information is accurate from): __________________________________________________________________________________

**Address and Location Information:**

First, we want to know what type of address and location data you may have within your cohort.

1. What is the process for collecting and updating participant addresses?

__________________________________________________________________________________

1. If not specified above, how often are participant residential addresses updated?

__________________________________________________________________________________

1. Do you have any of the following administrative geographic data on participants’ residential addresses (including collected from surveys/participant self-report)? Check all that apply:
   - ZIP code or ZCTA
   - County
   - City
   - State
   - Other ____________________________
2. Do you have any participant residential addresses geocoded or georeferenced (i.e. the process of calculating corresponding geographic coordinates for a location)?

- Yes 🡪 Continue to Q5
- No 🡪 Skip to question 11
- Do not know🡪 Skip to question 11

Who may be better able to tell us whether these exist: __________________________

1. How frequently have participant addresses been geocoded?
   - Once (cross-sectionally)🡪

Please indicate what time/year geocoded addresses correspond to:

_______________________________________________________

- - Multiple times (longitudinally)🡪
    - - Entire duration of the study
      - Other, please specify: ____________________________________________________
  - Don’t Know

1. What level are participant addresses geocoded to?
   - Latitude/longitude
   - ZIP+4 or ZIP code
   - Both
   - Other
   - Don’t Know
2. Which administrative units are attached to participant geocodes? Check all that apply:
   - ZIP+4
   - Census block
   - Census block group
   - Census tract
   - MSA
   - ZCTA
   - Other, please specify:______________
3. Were buffers created around participant addresses?

- No
- Yes, Euclidean (straight-line), please specify distances: ________________________________
  - Yes, network (street), please specify distances: ______________________________________
  - Other, please specify: __________________________________________________________
  - Don’t Know

1. Did the level of geocoding (e.g., latitude/longitude versus zip code) change over time so that one level is available some years and another is available other years?
   - Yes
   - No
2. If known, what software and underlying street line files were used to georeference/geocode the addresses: _________________________________________________________________________

__________________________________________________________________________________

1. Did [study name] collect additional geographic locations beyond residential address (e.g. work address, volunteer location, secondary homes)
   - Yes🡪
     - What additional locations? _______________________________________________
     - Are they geocoded?
       - Yes
       - No
       - Do not know
   - No

**Neighborhood Level Data:**

Next, we want to know the details of neighborhood level data (both GIS- and survey-based) you may have within your cohort. Please indicate if neighborhood level data is available and details of these data. This is meant to be comprehensive and may include many categories of data that are not currently calculated and linked to participant addresses in your cohort.

| **Natural environment** | **Yes/**  **No** | **Metric(s)** | **Source** (e.g., survey based, GIS based, audit) | **Specific data source and year, if known** | **Level(s) of measurement (**e.g. buffer, zip code, Census tract, self-reported neighborhood) | **Timing of measurement** (year(s) of residence metric applies to) | **Additional Notes** |
| --- | --- | --- | --- | --- | --- | --- | --- |
| Green space |  |  |  |  |  |  |  |
| Blue space/water bodies |  |  |  |  |  |  |  |
| Temperature |  |  |  |  |  |  |  |
| Weather |  |  |  |  |  |  |  |
| Air quality or pollution |  |  |  |  |  |  |  |
| Light at night |  |  |  |  |  |  |  |
| Hilliness/elevation |  |  |  |  |  |  |  |
| Noise |  |  |  |  |  |  |  |
| Other (please specify) |  |  |  |  |  |  |  |

| **Built environment** | **Yes/**  **No** | **Metric(s)** | **Source** (e.g., survey based, GIS based, audit) | **Specific data source and year, if known** | **Level(s) of measurement (**e.g. buffer, zip code, Census tract, self-reported neighborhood) | **Timing of measurement** (year(s) of residence metric applies to) | **Additional Notes** |
| --- | --- | --- | --- | --- | --- | --- | --- |
| Healthy and unhealthy food store access (e.g., supermarkets) |  |  |  |  |  |  |  |
| Alcohol outlets |  |  |  |  |  |  |  |
| Tobacco/marijuana outlets |  |  |  |  |  |  |  |
| Recreation/physical activity facilities |  |  |  |  |  |  |  |
| Commercial buildings |  |  |  |  |  |  |  |
| Housing density |  |  |  |  |  |  |  |
| Retail use/stores |  |  |  |  |  |  |  |
| Land use mix/entropy |  |  |  |  |  |  |  |
| Social destinations (those for spending time with others e.g. bars, libraries, churches) |  |  |  |  |  |  |  |
| Walking destinations (those needed for life on a weekly basis e.g. banks) |  |  |  |  |  |  |  |
| Walkability (composite measure) |  |  |  |  |  |  |  |
| Access to health care facilities |  |  |  |  |  |  |  |
| Street connectivity/intersection density |  |  |  |  |  |  |  |
| Public Transportation (e.g., bus, rail) |  |  |  |  |  |  |  |
| Traffic/closest major road |  |  |  |  |  |  |  |
| Sidewalks or Bike Infrastructure |  |  |  |  |  |  |  |
| Aesthetics |  |  |  |  |  |  |  |
| Accessibility Amenities (benches, lights, sidewalk cuts, sidewalk crossings) |  |  |  |  |  |  |  |
| Other (please specify) |  |  |  |  |  |  |  |

| **Social environment** | **Yes/**  **No** | **Metric(s)** | **Source** (e.g., survey based, GIS based, audit) | **Specific data source and year, if known** | **Level(s) of measurement (**e.g. buffer, zip code, Census tract, self-reported neighborhood) | **Timing of measurement** (year(s) of residence metric applies to) | **Additional Notes** |
| --- | --- | --- | --- | --- | --- | --- | --- |
| Census measures: Socioeconomic status |  |  |  |  |  |  |  |
| Census measures: Housing or rentals |  |  |  |  |  |  |  |
| Census measures:  Nativity or foreign born |  |  |  |  |  |  |  |
| Census measures: Racial/ethnicity |  |  |  |  |  |  |  |
| Calculated Measures: Racial/ethnic segregation or ethnic enclaves |  |  |  |  |  |  |  |
| Census Measures: Population counts/Age distributions |  |  |  |  |  |  |  |
| Urbanicity (e.g. RUCA, MSA) |  |  |  |  |  |  |  |
| Pedestrian/bike crashes |  |  |  |  |  |  |  |
| Vehicular crashes |  |  |  |  |  |  |  |
| Violence or crime |  |  |  |  |  |  |  |
| Social engagement |  |  |  |  |  |  |  |
| Social support or engagement (including social cohesion and trust) |  |  |  |  |  |  |  |
| Foreclosures |  |  |  |  |  |  |  |
| Trash/litter/graffiti/broken windows (i.e., “disorder”) |  |  |  |  |  |  |  |
| Crowding within households |  |  |  |  |  |  |  |
| Political power |  |  |  |  |  |  |  |
| Policies related to health (e.g., sugar tax laws, zoning codes, age-friendly initiatives) |  |  |  |  |  |  |  |
| Other (please specify) |  |  |  |  |  |  |  |

| **COVID neighborhood measures** | **Yes/No** | **Metric(s)** | **Source (e.g., survey based, reported by government/ agency, other)** | **Level(s) of measurement (e.g. city-level, zip code, Census tract, neighborhood)** | **Timing of measurement (years, months, quarters?)** | **Additional Notes** |
| --- | --- | --- | --- | --- | --- | --- |
| Number of cases |  |  |  |  |  |  |
| Number of deaths |  |  |  |  |  |  |
| Vaccination rates |  |  |  |  |  |  |
| Local disparity indices (in any of the above) |  |  |  |  |  |  |
| Policies (e.g., lockdown measures, etc.) |  |  |  |  |  |  |
| Other (please specify) |  |  |  |  |  |  |

**Any additional comments or information we have not covered?** __________________________________________________________________________________________________________________

____________________________________________________________________________________________________________________________________________________________________________________________________________________________________

__________________________________________________________________________________________________________________

__________________________________________________________________________________________________________________

__________________________________________________________________________________________________________________

__________________________________________________________________________________________________________________

__________________________________________________________________________________________________________________

**Thank you again for your time and effort!**
